# A Multicenter Propensity Score-Matched Cohort Study of Preoperative Antiplatelet Therapy and Postoperative Outcomes in Elderly Surgical Patients

**DOI:** 10.3390/medicina62030521

**Published:** 2026-03-11

**Authors:** Seokyoung Song, Hyungseok Seo, Il Seok Kim, Minsoo Kim, Lim Youn Hee, Jung Eun Kim, Soo Il Choi, Dong Hyuck Kim, Young Hun Lee, Moonki Park, Jong Bum Choi, Cheolhyeong Lee, Seung Hee Yoo, Ho Kyung Yu, Chan Noh, Seong Young Choi, Sang Gyu Kwak

**Affiliations:** 1 Department of Anesthesiology and Pain Medicine, Daegu Catholic University School of Medicine, Daegu 42472, Republic of Korea; anessy73@gmail.com (S.S.); eastokim@gmail.com (D.H.K.); hyo04076@gmail.com (Y.H.L.); 2 Department of Anesthesiology and Pain Medicine, Kyung Hee University Hospital at Gangdong, Seoul 05278, Republic of Korea; seohyungseok@gmail.com; 3 Department of Anesthesiology and Pain Medicine, Kangdong Sacred Heart Hospital, Hallym University College of Medicine, Seoul 05355, Republic of Korea; gns70@kdh.or.kr; 4 Department of Anesthesiology and Pain Medicine, Kangwon National University Hospital, College of Medicine, Kangwon National University, Chuncheon 24289, Republic of Korea; kmsanp@kangwon.ac.kr; 5 Department of Anesthesiology and Pain Medicine, Gyeongsang National University Hospital, Jinju 52727, Republic of Korea; im6194@naver.com; 6 Department of Anesthesiology and Pain Medicine, Kyung Hee University Hospital, Kyung Hee University College of Medicine, Seoul 02447, Republic of Korea; geri200@gmail.com; 7 Department of Anesthesiology and Pain Medicine, International St. Mary’s Hospital, Incheon 22711, Republic of Korea; swiri31@naver.com; 8 Department of Anesthesiology and Pain Medicine, Myongji Hospital, Goyang 10474, Republic of Korea; psbuster@naver.com; 9 Department of Anesthesiology and Pain Medicine, Ajou University Medical Center, Suwon 16477, Republic of Korea; romeojb@naver.com; 10 Department of Anesthesiology and Pain Medicine, Wonkwang University School of Medicine, Iksan 54538, Republic of Korea; leecheolhyeong@gmail.com; 11 Department of Anesthesiology and Pain Medicine, Ewha Womans University Mokdong Hospital, Seoul 07985, Republic of Korea; yoosh0710@naver.com; 12 Department of Anesthesiology and Pain Medicine, Gyeongsang National University Changwon Hospital, Changwon 51472, Republic of Korea; sciatic@naver.com; 13 Department of Anesthesiology and Pain Medicine, College of Medicine, Chungnam National University, Daejeon 35015, Republic of Korea; jyfchrh@naver.com; 14 EvidNet, Gangnam-gu, Seoul 06164, Republic of Korea; tjddud2830@evidnet.com; 15 Department of Medical Statistics, School of Medicine, Daegu Catholic University, Duryugongwon-Ro 17-Gil 33, Nam-Gu, Daegu 42472, Republic of Korea

**Keywords:** antiplatelet therapy, elderly surgery, postoperative bleeding, cardiovascular events, propensity score matching, OMOP-CDM

## Abstract

*Background and Objectives*: Elderly patients frequently receive antiplatelet therapy, creating a clinical dilemma between bleeding risk and cardiovascular protection during surgery. We evaluated the association between preoperative antiplatelet therapy and postoperative bleeding and cardiovascular events using multicenter observational data. *Materials and Methods*: We conducted a retrospective cohort study using standardized OMOP-CDM databases from 10 tertiary hospitals. Patients aged ≥65 years undergoing surgery were classified by preoperative aspirin or clopidogrel exposure. Propensity score matching was performed within each site. Hazard ratios (HRs) were estimated using Cox regression and pooled using meta-analytic techniques. *Results*: A total of 1464 exposed patients and 7038 matched comparators were analyzed. Across sites, hazard ratios varied without a statistically significant pooled association. The pooled HR for postoperative events was 1.01 (95% CI 0.57–1.78, *p* = 0.967). Covariate balance improved substantially after matching. *Conclusions*: Preoperative antiplatelet therapy was not associated with a consistent increase in postoperative bleeding or cardiovascular events in elderly surgical patients. These findings support individualized perioperative management rather than routine discontinuation.

## 1. Introduction

Elderly patients represent a rapidly expanding proportion of the global surgical population. With increasing age, the prevalence of cardiovascular and cerebrovascular disease rises substantially, resulting in widespread long-term use of antiplatelet therapy for secondary prevention. Clinical practice guidelines emphasize careful perioperative cardiovascular risk assessment and management in patients undergoing non-cardiac surgery, particularly among those receiving antithrombotic therapy [1,2]. Antiplatelet agents such as aspirin and P2Y12 inhibitors play a central role in reducing thrombotic events in patients with established cardiovascular disease. However, their perioperative management remains controversial. Because antiplatelet agents and anticoagulants differ substantially in their mechanisms of action, clinical indications, and perioperative management strategies, the present study focused specifically on antiplatelet therapy to address a more clinically homogeneous exposure group. Continuation of antiplatelet therapy may increase the risk of perioperative bleeding, whereas discontinuation may expose vulnerable patients to myocardial infarction, stroke, or other thrombotic complications [3,4]. The perioperative management of antithrombotic therapy requires careful balancing of bleeding and thrombotic risks, and clinical decisions regarding continuation or interruption often depend on both the type of surgical procedure and the underlying cardiovascular indication for treatment [5].

Randomized trials evaluating perioperative aspirin therapy have reported heterogeneous findings. In the POISE-2 trial, perioperative aspirin did not reduce the risk of death or myocardial infarction but was associated with an increased risk of major bleeding [6]. Similarly, other randomized investigations examining perioperative cardiovascular interventions have highlighted the complex balance between ischemic and bleeding risks in surgical patients [7]. Observational studies have also suggested that perioperative cardiovascular and bleeding outcomes are influenced by multiple patient-level factors, including age, comorbidity burden, and perioperative management strategies [8,9,10,11]. These considerations are particularly relevant in elderly populations, who frequently present with multimorbidity, frailty, and polypharmacy and therefore face higher risks of both thrombotic and hemorrhagic complications during the perioperative period [12].

Despite the growing clinical importance of this issue, high-quality evidence specifically focused on elderly surgical patients remains limited. Traditional randomized trials often underrepresent older adults and may not fully capture real-world perioperative practice patterns. Elderly patients represent a particularly high-risk surgical population characterized by multimorbidity, frailty, and polypharmacy, yet they remain underrepresented in many randomized perioperative trials. Therefore, focusing specifically on elderly patients may provide clinically relevant evidence for perioperative decision-making in this vulnerable population.

Large-scale observational data sources therefore provide valuable opportunities to evaluate medication safety and clinical outcomes in routine clinical settings. Standardized data models such as the Observational Medical Outcomes Partnership (OMOP) common data model enable harmonized analyses across multiple institutions while preserving local data governance and reproducibility [13,14,15]. In addition, modern causal inference approaches, including propensity score methods, facilitate more rigorous control of confounding in observational studies [16,17,18].

Therefore, we conducted a multicenter retrospective cohort study using standardized OMOP common data model databases to evaluate the association between preoperative antiplatelet therapy and postoperative bleeding and cardiovascular events in elderly surgical patients. We hypothesized that antiplatelet exposure would not produce a uniform increase in adverse postoperative outcomes when observable confounding factors are rigorously controlled. By leveraging harmonized real-world data from multiple hospitals, this study aims to provide clinically relevant evidence to inform perioperative decision-making in older adults.

## 2. Methods

### 2.1. Study Design and Data Framework

This multicenter retrospective cohort study was conducted using electronic health record databases standardized to the Observational Medical Outcomes Partnership (OMOP) Common Data Model (CDM). Ten tertiary-care teaching hospitals in the Republic of Korea participated in a distributed research network. All participating institutions are large academic medical centers with established electronic health record systems and implemented OMOP-CDM infrastructures. Within this network, each institution executed identical analytic scripts locally and shared only aggregate results. No patient-level data were transferred across institutions, ensuring compliance with privacy regulations while maintaining methodological consistency.

Data transformation into the OMOP-CDM followed institutional quality control procedures, including vocabulary mapping validation and data completeness checks. The study adhered to the Strengthening the Reporting of Observational Studies in Epidemiology (STROBE) recommendations and the OHDSI best-practice framework for observational research.

The study protocol was approved by the Institutional Review Board of Daegu Catholic University Medical Center (DCUMC 2025-09-048). Informed consent was waived due to the retrospective analysis of de-identified data.

Generative AI (ChatGPT, OpenAI, version GPT-5.3) was used only for language editing and clarity improvement. The tool assisted in grammar polishing and stylistic refinement of the manuscript text. No AI tools were used for data analysis, interpretation of results, or scientific decision-making. All scientific content, study design, and conclusions were developed entirely by the authors, who take full responsibility for the manuscript.

### 2.2. Study Population

Eligible patients were identified from participating OMOP-CDM databases and included individuals aged 65 years or older who underwent inpatient surgical procedures requiring general anesthesia or major regional anesthesia between 1 January 2020 and 31 December 2024. The index date was defined as the date of surgery. To ensure adequate assessment of baseline characteristics, patients were required to have at least 365 days of observable medical history prior to the index date. A minimum follow-up period of 30 days after surgery was required unless death occurred earlier.

Patients were excluded if they experienced a major bleeding event or an acute cardiovascular event, including myocardial infarction or ischemic stroke, within 30 days before surgery. Additional exclusion criteria included death on the day of surgery, incomplete prescription or outcome records, and insufficient baseline observation time. Eligible patients were followed from the index date until occurrence of an outcome event, death, loss to follow-up, or 30 days after surgery, whichever occurred first. The study period (1 January 2020 to 31 December 2024) was selected to include the most recent five years with harmonized OMOP-CDM data available across all participating institutions, ensuring consistent data structure, coding practices, and sufficient cohort size for multicenter analysis.

### 2.3. Exposure Definition

The target cohort consisted of patients with documented prescription records for aspirin or clopidogrel within 30 days prior to surgery. Exposure classification was based exclusively on outpatient or inpatient prescription data captured in the OMOP drug exposure table. Patients with any qualifying prescription within the exposure window were classified as exposed. The comparator cohort included patients with no antiplatelet prescription during the same preoperative period. Exposure status was fixed at baseline and analyzed under an intention-to-treat framework. Aspirin and clopidogrel were analyzed as a combined antiplatelet exposure to reflect real-world perioperative decision-making and to preserve statistical power. Agent-specific analyses were not performed because the number of outcome events was limited across institutions.

### 2.4. Outcome Definitions

The primary outcome was a composite of postoperative bleeding or cardiovascular events occurring within 30 days after surgery. Outcomes were identified using standardized condition concept sets within the OMOP-CDM framework and were applied uniformly across all participating institutions. Postoperative bleeding events included gastrointestinal bleeding, intracranial hemorrhage, and clinically significant surgical-site hematoma. Cardiovascular events included acute myocardial infarction, ischemic stroke, and systemic thromboembolism. These outcomes were selected to reflect clinically meaningful complications representing the principal safety concerns associated with perioperative antiplatelet therapy. All outcome definitions were based on diagnosis records mapped to standardized OMOP condition concepts. When available, previously validated phenotype definitions were used to enhance reproducibility and minimize misclassification. Identical outcome algorithms were implemented at each institution to ensure consistency across sites. Outcome ascertainment was standardized across institutions using identical phenotype definitions and analytic specifications to minimize inter-institutional variability.

### 2.5. Covariate Assessment and Propensity Score Matching

Baseline covariates were assessed during the 365-day period preceding surgery and included demographic characteristics, comorbidities, medication exposures, and healthcare utilization markers. Comorbidities were identified using standardized condition concepts. Medication covariates included antihypertensives, anticoagulants, lipid-lowering agents, antidiabetics, nonsteroidal anti-inflammatory drugs, and other clinically relevant drug classes. A high-dimensional covariate set was constructed without outcome-based variable selection to minimize confounding.

Propensity scores predicting antiplatelet exposure were estimated separately within each institution using logistic regression models including all baseline covariates. Nearest-neighbor matching without replacement was performed using a caliper width of 0.2 standard deviations of the logit of the propensity score. Matching ratios varied by institution depending on available cohort sizes to optimize balance while preserving sample size. Covariate balance was assessed using standardized mean difference (SMD), with values < 0.1 considered acceptable.

### 2.6. Statistical Analysis

All statistical analyses were conducted using a distributed analytic approach based on the OHDSI framework. Identical analysis scripts were executed independently at each participating institution, and only aggregated, de-identified summary statistics were shared for central synthesis. For each institution, time-to-event analyses were performed to estimate the association between preoperative antiplatelet therapy and the primary composite outcome. Follow-up time was calculated from the index date (date of surgery) until the occurrence of the outcome event, death, loss to follow-up, or 30 days after surgery, whichever occurred first. Hazard ratios (HRs) and corresponding 95% confidence intervals (CIs) were estimated using Cox proportional hazards regression models applied to the propensity score-matched cohorts. The proportional hazards assumption was assessed descriptively where feasible, and no obvious violations were observed. Given the relatively low number of outcome events at several institutions, model complexity was intentionally limited to preserve estimation stability. Site-specific effect estimates were pooled using a random-effects meta-analysis to account for between-site variability arising from differences in patient populations and institutional practices. The DerSimonian–Laird method was used to estimate the between-site variance. Overall pooled HRs and 95% CIs were reported as the primary summary measures. Statistical heterogeneity across institutions was evaluated qualitatively by comparing point estimates and confidence intervals across sites and was summarized quantitatively using the I^2^ statistic. Forest plots were generated to visually display institution-specific and pooled effect estimates. All analyses were conducted using R statistical software, version 4.3.2 within the OHDSI analytic pipeline. Statistical significance was defined as a two-sided *p*-value less than 0.05. The full reproducible cohort design and analytic specifications are publicly available at “https://github.com/sanggyu3939/antiplatelet-perioperative-study (accessed on 12 February 2026)” to support open science and independent validation. Detailed cohort definitions and executable analytic specifications are publicly available in the study GitHub repository, including the JSON files *target_cohort.json*, *comparator_cohort.json*, *outcome_definitions.json*, and *estimation.json*. Site-specific analytic outputs included cohort counts, covariate balance summaries, and hazard ratio estimates with corresponding confidence intervals, which were then centrally synthesized without transfer of patient-level data. This distributed approach ensured methodological consistency while preserving institutional data privacy.

## 3. Results

### 3.1. Cohort Construction

**Figure 1:**
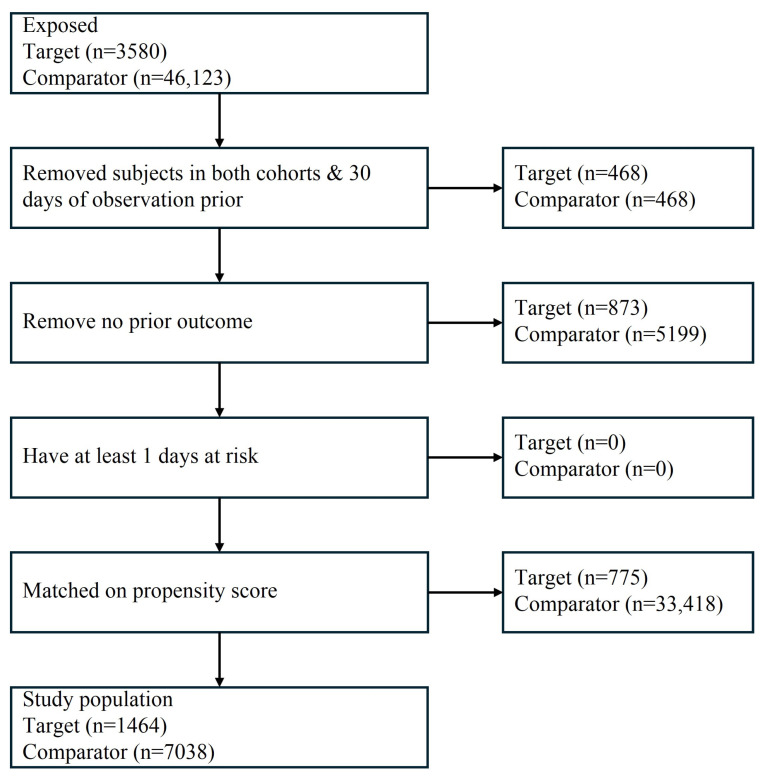
Cohort construction and propensity score matching workflow.

The cohort construction process is illustrated in Figure 1. Across the participating OMOP-CDM databases, a total of 49,703 elderly patients undergoing eligible surgical procedures were initially identified, including 3580 patients with preoperative antiplatelet prescriptions and 46,123 comparator patients without documented exposure. After applying inclusion and exclusion criteria and performing site-specific propensity score matching, 1464 exposed patients and 7038 matched comparator patients were included in the final analytic cohort.

Matching ratios varied across institutions according to available cohort sizes and baseline characteristics. The distributed matching approach preserved local data governance while maintaining analytic consistency across sites.

### 3.2. Covariate Balance

Substantial baseline imbalance was observed before propensity score adjustment, particularly in cardiovascular comorbidities and medication exposures associated with antiplatelet therapy. Median absolute standardized mean differences exceeded 0.5 for several clinically important variables prior to matching, indicating marked differences between exposed and comparator populations.

After matching, covariate balance improved considerably across institutions, and most covariates achieved acceptable balance with median absolute standardized mean differences below 0.1. A small number of cardiovascular medication variables remained modestly imbalanced (standardized mean difference approximately 0.2), likely reflecting persistent co-prescription patterns and the strong clinical linkage between antiplatelet therapy and underlying cardiometabolic risk in elderly patients. Residual imbalance did not demonstrate a consistent directional pattern across sites.

**Figure 2:**
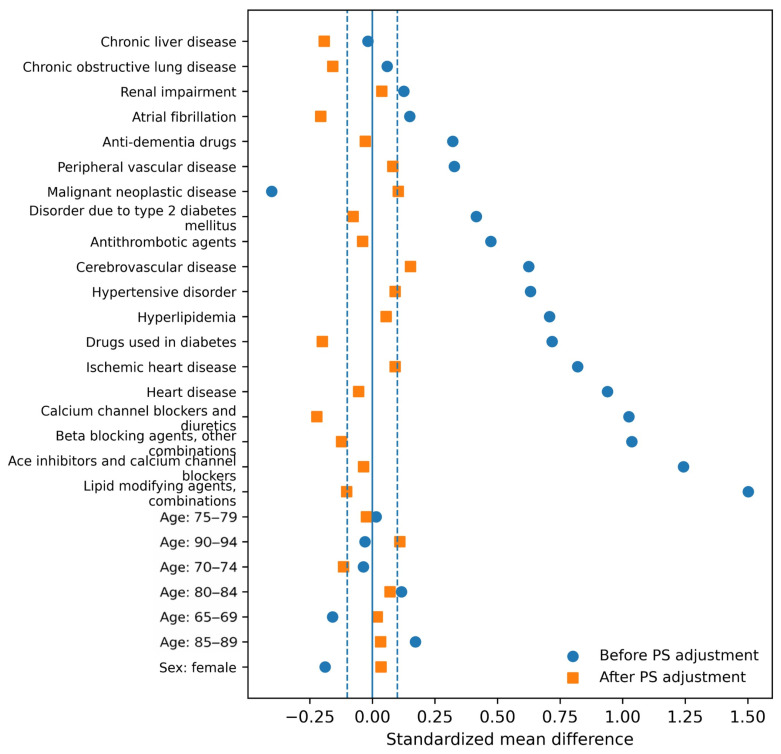
Covariate balance before and after propensity score adjustment.

Figure 2 and Table 1 summarize covariate balance before and after matching and confirm substantial reduction in observable confounding across institutions. Before matching, the greatest imbalance was observed in cardiovascular comorbidities and cardiometabolic medication use, including heart disease, ischemic heart disease, statin use, and antihypertensive treatment. After matching, most covariates were substantially improved, although modest residual imbalance remained in selected cardiovascular medication variables. These findings suggest that propensity score matching meaningfully reduced observable confounding while preserving a clinically relevant elderly surgical cohort.

### 3.3. Postoperative Outcomes

Within 30 days after surgery, 50 composite adverse events occurred among 1464 exposed patients and 87 events occurred among 7038 matched comparator patients, corresponding to crude event proportions of 3.4% and 1.2%, respectively. These crude proportions are presented for descriptive context only; primary inference was based on propensity score-matched time-to-event analyses that account for censoring and variable follow-up. Although crude event proportions differed numerically between groups, inference was based on matched time-to-event models because crude comparisons do not account for censoring and remaining differences in baseline risk. Overall event rates were low in both groups, consistent with the short follow-up period and the relative infrequency of severe postoperative bleeding or cardiovascular complications in contemporary perioperative care.

Institution-specific hazard ratio estimates varied, but confidence intervals were wide at several sites due to sparse event counts. In many institutions, fewer than ten outcome events were observed per exposure group, limiting statistical precision. No individual institution demonstrated a statistically significant increase or decrease in risk associated with preoperative antiplatelet exposure.

**Figure 3:**
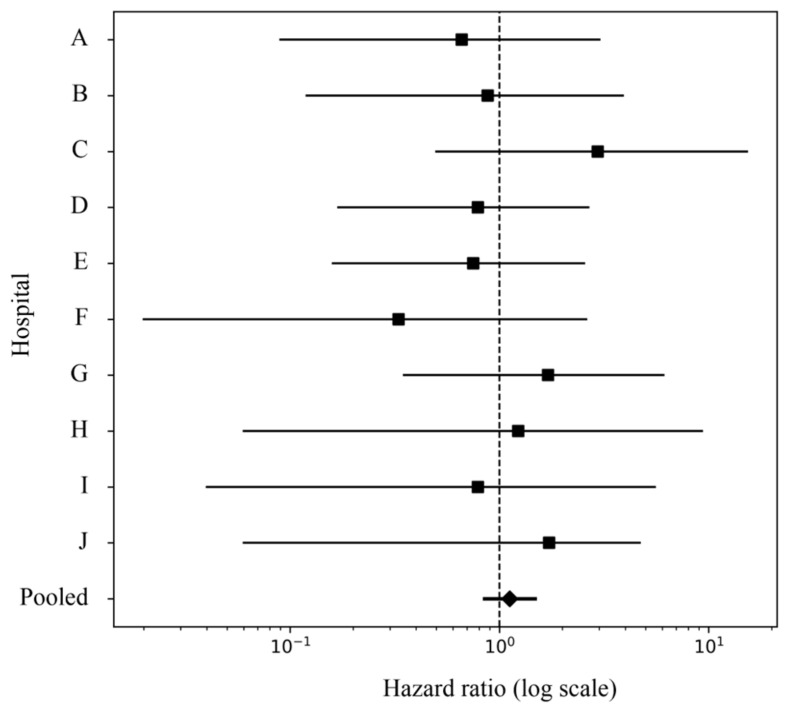
Forest plot of site-specific and pooled hazard ratios for the primary outcome. Squares represent hazard ratio estimates for each hospital (Sites A–J), and horizontal lines indicate 95% confidence intervals. The diamond represents the pooled hazard ratio from the random-effects meta-analysis. The dashed vertical line indicates the null value (hazard ratio = 1).

The pooled hazard ratio from the random-effects meta-analysis was 1.01 (95% CI 0.57–1.78; *p* = 0.967), centered near the null value and compatible with both modest risk reduction and modest risk increase. The absence of a consistent directional pattern across institutions suggests that observed variability primarily reflects random fluctuation rather than systematic differences in perioperative practice. Between-site heterogeneity was negligible (I^2^ = 0%), indicating highly consistent effect estimates across institutions. Forest plots are presented in Figure 3.

Table 2 summarizes site-level cohort sizes, event rates, and hazard ratio estimates. The number of matched exposed patients ranged from 8 to 342 across hospitals. Outcomes were infrequent at most sites, resulting in wide confidence intervals. Estimates from Site F should be interpreted cautiously due to the extremely small matched sample size. Despite variation in point estimates, no institution showed a statistically significant association, consistent with the pooled meta-analytic findings.

## 4. Discussion

This multicenter propensity score-matched cohort study using harmonized OMOP-CDM databases found no evidence of a consistent association between preoperative antiplatelet therapy and postoperative bleeding or cardiovascular events in elderly surgical patients. The pooled effect estimate was centered near the null value, and between-site heterogeneity was negligible, suggesting that the absence of a strong association was consistent across institutions rather than driven by a single site. These findings indicate that antiplatelet exposure alone may not uniformly translate into adverse perioperative outcomes when observable confounding is carefully addressed.

The perioperative management of antiplatelet therapy remains a persistent clinical dilemma, particularly in older adults who face competing risks of hemorrhage and thrombosis [1,3,4]. Randomized trials evaluating perioperative aspirin therapy have reported heterogeneous findings. In the POISE-2 trial, perioperative aspirin did not reduce the risk of death or myocardial infarction but increased the risk of major bleeding [6]. Other randomized investigations examining perioperative cardiovascular interventions have similarly highlighted the complex balance between ischemic and bleeding risks in surgical patients [7]. Observational studies have also reported variable associations between perioperative antiplatelet exposure and clinical outcomes, reflecting differences in patient populations, surgical characteristics, and perioperative management strategies [8,9,10,11].

Previous studies have also highlighted that the decision to continue or interrupt antithrombotic therapy during surgery often depends on procedure-specific bleeding risk and the patient’s underlying cardiovascular condition [5]. Some prospective studies have suggested that continuation of antithrombotic therapy may be feasible in selected low-risk surgical procedures without substantially increasing postoperative bleeding complications.

Older adults represent a particularly vulnerable surgical population. Multimorbidity, frailty, and polypharmacy are highly prevalent in elderly patients and may amplify both thrombotic and bleeding risks during the perioperative period [12]. However, elderly patients are frequently underrepresented in randomized trials, limiting the generalizability of trial-based evidence to routine clinical practice. By focusing specifically on patients aged 65 years or older and leveraging multi-institutional real-world data, our study provides additional evidence addressing this important gap in perioperative research.

From a clinical perspective, our findings support individualized perioperative decision-making rather than routine discontinuation of antiplatelet therapy based solely on age. Contemporary perioperative care includes advances in surgical hemostasis, anesthesia techniques, and postoperative monitoring, which may mitigate bleeding risks that historically prompted routine discontinuation strategies [1,3]. At the same time, interruption of antiplatelet therapy may increase the risk of thrombotic complications in high-risk patients [4,10]. Our results therefore reinforce current guideline recommendations that emphasize balancing bleeding and ischemic risks within the broader clinical context of each patient [1,2]. In routine clinical practice, decisions regarding continuation or interruption of antiplatelet therapy before surgery are typically guided by procedure-specific bleeding risk, the underlying cardiovascular indication for therapy, and guideline-based recommendations. However, detailed information regarding these clinical decision-making processes was not available in the present retrospective database.

A notable strength of this study is the distributed observational framework enabled by the OMOP common data model. Standardized data harmonization allowed identical analytic protocols to be executed across institutions while preserving local data governance. This approach enhances reproducibility and external validity and enables evaluation of treatment safety across diverse healthcare environments [13,14,15]. In addition, the use of propensity score methods improves the ability to address confounding in observational data and strengthens the validity of causal inference in real-world research settings [16,17,18].

Several limitations should be acknowledged. First, residual confounding inherent to observational research cannot be completely eliminated despite rigorous propensity score adjustment [18]. Second, event counts were relatively low at several institutions, resulting in limited statistical precision and wide confidence intervals. Third, outcome definitions relied on administrative coding and may therefore be subject to misclassification. Fourth, detailed surgical characteristics, including procedure-specific bleeding risk categories, medication adherence, timing of drug discontinuation, and perioperative management strategies were not uniformly captured within the CDM structure. Surgical procedures in the cohort were heterogeneous across multiple specialties, and procedure-specific stratification was not performed in the present analysis. These factors may influence perioperative risk but could not be fully evaluated in the present analysis.

An important interpretive consideration is that a null association in observational perioperative research does not necessarily imply the absence of individual-level risk. Rather, it suggests that, at the population level, antiplatelet exposure alone may not be the dominant determinant of adverse postoperative outcomes when modern perioperative care is applied. Elderly surgical patients represent a heterogeneous population in which procedural risk, frailty, comorbidity burden, and perioperative management likely interact in complex ways [12]. Our findings therefore support a risk-stratified framework in which antiplatelet therapy is evaluated in conjunction with surgical bleeding risk, cardiovascular vulnerability, and patient-specific clinical priorities rather than as an isolated exposure.

From a methodological standpoint, the use of a harmonized distributed observational network addresses a central challenge in perioperative research: the difficulty of conducting adequately powered randomized trials in older adults. Observational data, when analyzed using transparent and reproducible frameworks, can complement randomized evidence by providing insight into real-world safety patterns across diverse clinical environments. The consistency of findings across independent institutions strengthens confidence that the absence of a strong signal is unlikely to be driven by single-center effects and may reflect broader patterns in contemporary perioperative practice.

Finally, these results highlight the evolving role of real-world evidence in perioperative medicine. As surgical populations continue to age, large-scale interoperable data infrastructures will become increasingly important for evaluating medication safety in groups traditionally excluded from trials. Integrating observational analytics into clinical guideline development may help bridge the gap between controlled trial environments and routine clinical care [1,13]. Importantly, our findings should not be interpreted as a recommendation to universally continue antiplatelet therapy but rather as evidence that routine discontinuation in elderly patients may not be justified without individualized risk assessment.

### Future Research Directions

Future research should incorporate procedure-specific stratification and more granular information on medication timing to better characterize risk in high-risk surgical subgroups. Prospective registries and pragmatic trials embedded within real-world data networks may further clarify the optimal perioperative management of antiplatelet therapy in aging populations. Continued development of standardized observational research infrastructures will be essential for generating reproducible evidence in populations that are traditionally underrepresented in clinical trials [13,14,15].

Taken together, the findings of this study provide contemporary multicenter evidence suggesting that preoperative antiplatelet therapy was not associated with a consistent increase in perioperative risk in elderly surgical patients. These findings support individualized risk assessment and highlight the value of large-scale harmonized data networks for evaluating medication safety in routine clinical practice.

## 5. Conclusions

In this multicenter propensity score-matched cohort study of elderly surgical patients, preoperative antiplatelet therapy was not associated with a consistent increase in postoperative bleeding or cardiovascular events. The pooled effect estimate centered near the null value across independent institutions, suggesting that antiplatelet exposure alone may not be a dominant determinant of perioperative risk in older adults when contemporary surgical and anesthetic care is applied.

These findings support an individualized approach to perioperative decision-making that balances bleeding and thrombotic risks within the broader clinical context rather than relying on routine discontinuation strategies based solely on age. While observational in nature, this study demonstrates the value of harmonized real-world data networks for evaluating medication safety in populations often underrepresented in randomized trials. Continued integration of standardized multi-institutional evidence will be essential for refining perioperative management strategies in aging surgical populations. These findings may help inform shared decision-making between surgeons, anesthesiologists, and patients when evaluating perioperative antiplatelet management.

## Figures and Tables

**Table 1:** Covariate balance before and after propensity-score adjustment.

Characteristic		|SMD| Before, Median (IQR)	|SMD| After, Median (IQR)
Age	65–69	0.21 (0.16–0.22)	0.08 (0.04–0.12)
	70–74	0.10 (0.04–0.12)	0.11 (0.04–0.19)
	75–79	0.06 (0.03–0.13)	0.03 (0.02–0.10)
	80–84	0.09 (0.06–0.12)	0.06 (0.03–0.07)
	85–89	0.04 (0.04–0.07)	0.11 (0.05–0.14)
Sex (female)		0.20 (0.12–0.27)	0.07 (0.04–0.10)
Medical history	CVD	0.34 (0.33–0.39)	0.12 (0.08–0.16)
	HD	0.83 (0.73–0.87)	0.23 (0.08–0.33)
	IHD	0.76 (0.71–0.90)	0.14 (0.10–0.25)
	PVD	0.42 (0.33–0.47)	0.05 (0.04–0.11)
	CLD	0.02 (0.02–0.04)	0.10 (0.07–0.12)
	DEM	0.20 (0.15–0.21)	0.08 (0.07–0.15)
	DM	0.31 (0.19–0.42)	0.08 (0.05–0.12)
	GERD	0.09 (0.07–0.15)	0.08 (0.07–0.08)
	HTN	0.66 (0.35–0.70)	0.16 (0.09–0.30)
	OA	0.08 (0.02–0.11)	0.08 (0.03–0.13)
	RI	0.30 (0.21–0.36)	0.11 (0.07–0.12)
	UTI	0.06 (0.04–0.15)	0.08 (0.02–0.11)
	Malignancy	0.26 (0.10–0.36)	0.07 (0.03–0.15)
	Colon CA	0.05 (0.03–0.14)	0.09 (0.08–0.12)
	Lung CA	0.08 (0.07–0.09)	0.14 (0.07–0.21)
Medication use	RAS inhibitors	0.70 (0.62–0.81)	0.04 (0.02–0.28)
	Antibiotics	0.12 (0.12–0.15)	0.05 (0.03–0.10)
	AD	0.14 (0.06–0.22)	0.10 (0.07–0.18)
	AED	0.17 (0.07–0.24)	0.07 (0.04–0.10)
	NSAIDs	0.09 (0.07–0.21)	0.04 (0.01–0.15)
	Anticancer agents	0.12 (0.01–0.15)	0.07 (0.04–0.08)
	Antithrombotics	0.56 (0.42–0.70)	0.14 (0.09–0.30)
	Beta-blockers	0.61 (0.48–0.67)	0.08 (0.05–0.18)
	CCB	0.66 (0.47–0.80)	0.13 (0.06–0.25)
	Diuretics	0.45 (0.25–0.56)	0.12 (0.07–0.30)
	Bronchodilators	0.12 (0.04–0.14)	0.07 (0.05–0.20)
	Antidiabetics	0.59 (0.42–0.62)	0.18 (0.09–0.22)
	Statins	1.21 (1.04–1.38)	0.21 (0.17–0.28)
	Opioids	0.16 (0.08–0.36)	0.10 (0.02–0.20)
	Psycholeptics	0.28 (0.10–0.45)	0.12 (0.03–0.18)
	Psychostimulants	0.25 (0.11–0.28)	0.11 (0.04–0.13)

**Table 2:** Site-specific cohort sizes, event rates, and hazard ratios.

Site	Eligible Cohort	Matched Cohort	Event Rate (%)	HR (95% CI)	*p* Value
	T/C	T/C	T/C		
A	330/4603	105/507	4.8/1.6	0.66 (0.09–3.01)	0.643
B	127/2181	53/266	9.4/3.0	0.88 (0.12–3.89)	0.885
C	419/4446	224/1130	2.2/0.4	2.95 (0.50–15.23)	0.215
D	596/8306	246/1120	2.0/1.3	0.79 (0.17–2.66)	0.737
E	711/9279	334/1574	1.5/1.0	0.75 (0.16–2.54)	0.683
F	50/142	8/8	62.5/62.5	0.33 (0.02–2.60)	0.372
G	939/10,564	342/1775	1.5/0.6	1.71 (0.35–6.08)	0.461
H	152/4344	70/420	7.1/1.2	1.23 (0.06–9.30)	0.872
I	166/1247	55/138	9.1/3.6	0.79 (0.04–5.53)	0.851
J	90/1011	27/100	18.5/5.0	1.73 (0.06–4.68)	0.747
Pooled	3580/46,123	1464/7038	3.4/1.2	1.01 (0.57–1.78)	0.967

Event rates are calculated within the matched cohorts over 30 days after surgery. HRs were estimated using Cox regression within each site and pooled using a random-effects meta-analysis. Site F reflects an extremely small matched cohort size and should be interpreted with caution.

## Data Availability

The data presented in this study are available on request from the corresponding author under ethical and legal restrictions related to patient confidentiality.

## Abbreviations

The following abbreviations are used in this manuscript:

C	Comparator
CA	Carcinoma
CCBs	Calcium-channel blockers
CI	Confidence interval
CLD	Chronic liver disease
CVD	Cerebrovascular disease
DEM	Dementia
DM	Diabetes mellitus
GERD	Gastroesophageal reflux disease
HD	Heart disease
HR	Hazard ratio
HTN	Hypertensive disorder
IHD	Ischemic heart disease
IQR	Interquartile range
NSAIDs	Nonsteroidal anti-inflammatory drugs
OA	Osteoarthritis
PVD	Peripheral vascular disease
RAS	Renin–angiotensin system
RI	Renal impairment
T	Target
UTI	Urinary tract infection
